# Implementation of Systematic Bioanalysis of Antibody–Drug Conjugates for Preclinical Pharmacokinetic Study of Ado-Trastuzumab Emtansine (T-DM1) in Rats

**DOI:** 10.3390/pharmaceutics15030756

**Published:** 2023-02-24

**Authors:** Eun-Jeong Jeon, Ju-Hee Han, Youjin Seo, Eun Mi Koh, Kang-Hyun Han, Kyunghwa Hwang, Kyung Jin Jung

**Affiliations:** 1 Immunotoxicology Research Group, Korea Institute of Toxicology, 141, Gajeong-ro, Yuseong-gu, Daejeon 34114, Chungcheongnam-do, Republic of Korea; 2 Jeonbuk Analytical Research Group, Jeonbuk Branch Institute, Korea Institute of Toxicology, 30, Baekhak 1-gil, Jeongeup-si 56212, Jeollabuk-do, Republic of Korea; 3 Department of Advanced Toxicology Research, Korea Institute of Toxicology, 141, Gajeong-ro, Yuseong-gu, Daejeon 34114, Chungcheongnam-do, Republic of Korea; 4 Jeonbuk Branch Institute, Korea Institute of Toxicology, 30, Baekhak 1-gil, Jeongeup-si 56212, Jeollabuk-do, Republic of Korea

**Keywords:** antibody–drug conjugate, ado-trastuzumab emtansine, bioanalysis, ELISA, mass spectrometry, immunogenicity, rat

## Abstract

Antibody–drug conjugates (ADCs) are composed of monoclonal antibodies covalently bound to cytotoxic drugs by a linker. They are designed to selectively bind target antigens and present a promising cancer treatment without the debilitating side effects of conventional chemotherapies. Ado-trastuzumab emtansine (T-DM1) is an ADC that received US FDA approval for the treatment of HER2-positive breast cancer. The purpose of this study was to optimize methods for the quantification of T-DM1 in rats. We optimized four analytical methods: (1) an enzyme-linked immunosorbent assay (ELISA) to quantify the total trastuzumab levels in all drug-to-antibody ratios (DARs), including DAR 0; (2) an ELISA to quantify the conjugated trastuzumab levels in all DARs except DAR 0; (3) an LC–MS/MS analysis to quantify the levels of released DM1; and (4) a bridging ELISA to quantify the level of anti-drug antibodies (ADAs) of T-DM1. We analyzed serum and plasma samples from rats injected intravenously with T-DM1 (20 mg/kg, single dose) using these optimized methods. Based on these applied analytical methods, we evaluated the quantification, pharmacokinetics, and immunogenicity of T-DM1. This study establishes the systematic bioanalysis of ADCs with validated assays, including drug stability in matrix and ADA assay, for future investigation on the efficacy and safety of ADC development.

## 1. Introduction

Antibody–drug conjugates (ADCs) are composed of monoclonal antibodies covalently bound to cytotoxic drugs (known as the payload) by a linker. ADCs specifically bind to targeted cell surface antigens overexpressed on tumor cells but minimally expressed on normal tissue [1,2]. The combination of the monoclonal antibody with the payload forms a complex molecular entity that combines the characteristics of a large-molecule biologic with those of a small-molecule drug [3,4]. Therefore, ADCs can be delivered selectively to tumor cells and have the high potency of cytotoxic drugs with minimal systemic toxicity [3,5]. ADCs are now one of the fastest growing drug classes for cancer therapy. To date, twelve ADCs have been approved by the U.S. Food and Drug Administration [6,7]. Of the twelve ADCs approved, five (i.e., Kadcyla^®^, Padcev^®^, Enhertu^®^, Trodelvy^®^, and Tivdak^®^) were approved for solid tumors. The others, Adcetris^®^, Besponsa^®^, Lumoxiti^®^, Mylotarg^®^, Polivy^®^, Blenrep^®^, and Zynlonta^®^, were approved for hematologic malignancies [6,7]. In addition, more than 80 ADCs are currently being evaluated in clinical trials [8].

The development of ADCs began with the research advances of binding monoclonal antibodies (mAbs) to highly specific targets on surface antigens in damaged or cancer cells [9,10,11,12]. Ado-trastuzumab emtansine (also called T-DM1 or trade name Kadcyla^®^) is an ADC that is used for the treatment of advanced breast cancers that are strongly positive for HER2 [13,14,15]. T-DM1 incorporates the HER2-targeted antitumor properties of trastuzumab with the cytotoxic activity of the microtubule-inhibitory agent DM1 (emtansine, a derivative of maytansine); the antibody and the cytotoxic agent are conjugated by a nonreducible thioether linker, *N*-succinimidyl-4-(*N*-maleimidomethyl)-cyclohexane-1-carboxylate (SMCC) [16,17]. This SMCC linker allows the conjugation between the lysine amines of antibodies and DM1, exhibiting a mixture with a drug-to-antibody ratio (DAR) range of 0 to 8 with an average DAR of approximately 3.5 [13,16]. The antibody backbone of T-DM1, trastuzumab (Herceptin^®^), which binds selectively to the extracellular domain of HER2 with a high affinity, was previously approved for the treatment of HER2-overexpressing breast cancer [17]. The payload of T-DM1, mertansine (called DM1), is a thiol-containing derivative of maytansine that can prevent microtubule assembly by inhibiting the polymerization of tubulin as a potent antimitotic agent [18,19]. Although the potential of maytansine as an anticancer drug was investigated in clinical trials, it was considered ineffective due to its systemic toxicity [20]. After the antibody-conjugatable maytansinoid was developed to overcome the systemic toxicity associated with maytansine and to raise tumor-specific delivery, DM1 was conjugated to trastuzumab to form T-DM1, which appeared safe and effective in patients [21]. The binding of T-DM1 to HER2 results in the entry of the HER2-T-DM1 complex into cells via receptor-mediated endocytosis. Because the linker is noncleavable and stable both in the circulation and in the tumor microenvironment, the release of DM1 occurs only as a result of the proteolysis of the antibody of T-DM1 in lysosomes [16]. After being released from lysosomes, DM1-containing metabolites inhibit microtubule assembly, eventually leading to cell death [22].

Despite the continued advancements in the field of ADC research, several challenges still need to be addressed. The fact that ADCs are complex mixtures converting high DAR species to low DAR species in vivo provokes fundamental challenges for pharmacokinetics (PK) bioanalysis [23]. Multiple and diverse analytical methods contribute to the challenges of ADC bioanalysis to understand the overall PK and toxicokinetics (TK) of an ADC [5,24]. Three fundamental assays used to assess the exposure and catabolism of most FDA-approved ADCs are total antibody, conjugated antibody, and unconjugated drugs [25]. As they have the molecular characteristics of both small- and large-molecule therapeutics, typical bioanalytical methods for both therapeutics are needed. For example, large molecules have well-defined tertiary structures suitable for ligand-binding assays (LBAs), such as an enzyme-linked immunosorbent assay (ELISA) [24]. Therefore, they can be used to measure the total antibody levels of an ADC at all DARs including DAR 0. ELISA can also be used to measure levels of conjugated antibodies to detect payload-attached antibodies using reagents that bind to the payload at all DARs except DAR 0. In addition, LC–MS/MS assays for small molecules need to be developed to measure unconjugated drugs. Moreover, ADCs are known to elicit anti-drug antibody (ADA) responses, similar to other biologics [26,27,28,29,30]. The ADAs are generated against all components of the intact ADC, which consists of a linker, payload, and unconjugated antibody. The proper detection of ADAs is also critical to evaluate their effect on efficacy, PK, and TK, which may influence the understanding of drug behaviors in preclinical experiments and later the understanding of treatment in patients.

**Figure 1:**
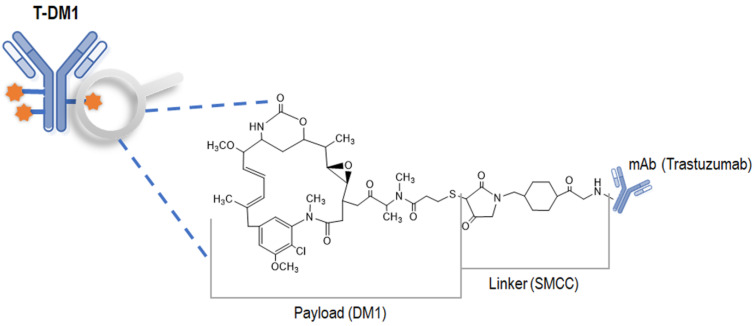
Chemical structure of T-DM1. T-DM1, a type of ADC, consists of the monoclonal antibody trastuzumab, a thioether linker SMCC, and the potent cytotoxic agent DM1 (also known as the payload). The payload linked in a part of T-DM1 is magnified.

In this study, all assays mentioned above were developed and optimized in rat serum/plasma to quantify each component of T-DM1, as shown in Figure 1: (1) ELISA for quantifying the total trastuzumab (all T-DM1 including unconjugated T-DM1) in the rat serum, (2) ELISA for quantifying the conjugated T-DM1 (all T-DM1 DARs except DAR 0) in the rat serum, (3) the LC–MS/MS assay for quantifying the free DM1 in the rat plasma, and (4) a bridging ELISA for an immunogenicity assessment of the T-DM1 in the rat serum. Herein, we describe optimized bioanalytical assays for the quantification of T-DM1 that were utilized to execute preclinical PK studies in rats.

## 2. Materials and Methods

### 2.1. Materials

N2’-Deacetyl-N2’-(3-mercapto-1-oxopropyl)-maytansine (Mertansine, DM1 compound) was obtained from Abcam (Cambridge, UK). Freund’s complete adjuvant (FCA) and Freund’s incomplete adjuvant (FIA) were obtained from Sigma–Aldrich (St. Louis, MO, USA). BSA-conjugated DM1 was prepared by Abfrontier (Seoul, Republic of Korea). Protein A and Protein G were purchased from GE Healthcare. T-DM1 (trade name Kadcyla^®^) was purchased from Dongwon Pharmaceutical (Daejeon, Republic of Korea) for laboratory research use. Human anti-trastuzumab, a positive control antibody, was purchased from Bio-Rad (Puchheim, Germany). HER2-ECD was obtained from Sino Biological Inc. (Beijing, China). Human anti-Fc-specific antibody conjugated to peroxidase was purchased from Thermo Fisher Scientific (Waltham, MA, USA). Biotin-conjugated Kadcyla and HER2-ECD were prepared by AbFrontier (Seoul, Republic of Korea). Streptavidin-HRP was purchased from BD Pharmingen^TM^ (San Diego, CA, USA). Carbonate-bicarbonate buffer, TMB substrate, and stop buffer were purchased from Sigma–Aldrich (St. Louis, MO, USA). A 1x PBS-T was purchased from Fluka (Buchs, Switzerland). Individual Crl:CD (SD) rat blank sera were obtained from BioChemed (Winchester, VA, USA).

### 2.2. Animals

Specific pathogen-free Crl:CD (SD) rats (male, 21 weeks old) were obtained from Orient Bio Inc. (Gyeonggi, Republic of Korea). Animal testing was performed in accordance with the guidelines of the American Association for the Accreditation of Laboratory Animal Care (AAALAC), and all procedures were approved by the Institutional Animal Care and Use Committee (IACUC) at the Korea Institute of Toxicology (Approval No.1709-0348).

To obtain anti-DM1 polyclonal antibodies (pAb), Crl:CD (SD) rats were immunized with subcutaneous injections of 100 μg of BSA-conjugated DM1 in FCA adjuvant. Booster doses of the same immunogen in FIA adjuvant were injected three times every two weeks. Two weeks after the last injection, serum samples were collected, and the IgG antibody titers were confirmed by ELISA.

To perform the single-dose PK study, T-DM1 was administered to rats (n = 8) by a single intravenous (IV) injection at a dose of 20 mg/kg. Serum and plasma samples were collected from the tail vein at predose (0 h) and 5 min, 1 h, 10 h, 24 h, 96 h, 192 h, and 360 h after IV administration for the quantification of conjugated and unconjugated trastuzumab by ELISA, the quantification of free DM1 by the LC–MS/MS assay, and the immunogenicity assessment of T-DM1.

### 2.3. ELISA Methods for DM1-Conjugated Trastuzumab and Total Trastuzumab

To quantify T-DM1, two different ELISA methods were developed considering the guidelines of ligand binding assays. For the quantification of DM1-conjugated trastuzumab except unconjugated trastuzumab (DAR 0), an anti-DM1 polyclonal antibody was used to capture site-specific DM1 labeled to T-DM1, and biotinylated HER2-ECD and streptavidin HRP were used to detect T-DM1. For the quantification of the total trastuzumab bearing both the DM1-conjugated and unconjugated trastuzumab, HER2-ECD was used to capture the total T-DM1 and anti-human IgG, and the Fc-specific antibody conjugated with HRP was used to detect the trastuzumab of the total T-DM1. Thereafter, the TMB-induced colorimetric signal was stopped to measure the optical density at 450 nm using a SpectraMax 190 microplate reader (Molecular Devices Inc., Sunnyvale, CA, USA).

### 2.4. LC–MS/MS Analysis for DM1

Unconjugated DM1 was extracted from rat plasma using a previously published method [13]. Briefly, 30 μL of rat plasma was reacted with 3 μL of 26 mM Tris (2-carboxylethyl) phosphine in 5 mM ammonium acetate at 37 °C for 10 min to reduce any disulfide bonds. The unconjugated DM1 was extracted by adding 200 µL of acetonitrile, and then it was centrifuged for 5 min. Then, 100 μL of the supernatant was reacted with N-ethylmaleimide (NEM) in dimethyl sulfoxide at 37 °C for 20 min to prevent dimerization and block any thiol groups. Prior to injecting DM1-NEM into the mass spectrometer, 50 μL of 200 nM maytansine in acetonitrile was added as an internal standard.

DM1-NEM was quantified via an API 5000 mass spectrometer (AB Sciex LLC, Canada) coupled with an Agilent 1260 HPLC system (Agilent Technologies, Inc., Santa Clara, CA, USA). The column used was an Atlantis dC18 column (3 µm, 2.1 mm × 50 mm, Waters Corp., Milford, MA, USA). Chromatographic separation was conducted with a gradient elution at a flow rate of 500 µL/min as follows: the mobile phase consisted of 5 mM ammonium acetate in a 0.1% formic acid aqueous solution as A and 5 mM ammonium acetate in acetonitrile: H_2_O (95:5) with 0.1% formic acid as B. Gradient elution was as follows: 20–98% B in 2 min, 98% B in 4 min, 20% B in 7 min. The mass spectrometric analysis of DM1-NEM was carried out in positive ion mode using a multiple reaction monitoring (MRM) transition of [845.4]^+^ to [485.3]^+^. The MRM transition for maytansine was [692.2]^+^ to [547.2]^+^.

### 2.5. Analysis of Immunogenicity

To measure the ADA responses of T-DM1, a bridging ELISA method was used with biotinylated T-DM1 and streptavidin HRP. Thereafter, the TMB-induced colorimetric signal was stopped to measure the optical density at 450 nm using a SpectraMax M3 microplate reader (Molecular Devices Inc.). To assess immunogenicity, screening assays were performed by detecting ADA binding to T-DM1 in the samples. To determine whether the sample was positive or not, the negative cutoff value was determined using the following equation: mean absorbance of negative control sample × normalization factor. The samples determined to be positive were subjected to a confirmatory assay to verify whether the ADAs were specific to T-DM1. The samples were treated with 1 mg/mL T-DM1, and the measured values were observed in comparison with the corresponding untreated samples.

### 2.6. Statistics

The data from the ELISA methods were collected and analyzed using SoftMax Pro version 5.4.1 software (Molecular Devices Inc.), and a logistic model (4-parameter) was used for data processing and curve fitting. The data from the LC–MS/MS methods were analyzed using Analyst^®^ 1.5, and a 1/x^2^-weighted linear regression model was used for data processing. The concentration–time curve was plotted using WinNonlin version 8.1.0 (Certara Inc., Princeton, NJ, USA). The PK parameters were calculated with noncompartmental analysis (NCA) models. All the graphs were generated using GraphPad Prism version 9.5.0 (GraphPad Software, San Diego, CA, USA).

## 3. Results

### 3.1. Validation of ELISA for DM1-Conjugated Trastuzumab and Total Trastuzumab

Considering the properties of T-DM1, two ELISA methods were developed to compare the quantification of selective DM-1-conjugated trastuzumab to nonselective total trastuzumab. During validation, typical parameters were evaluated by both ELISA methods (Table 1). The results are provided in the supplemental information, Supplementary Material Tables S1–S6. The standard curve range for both ELISAs was 100.0 ng/mL–6000.0 ng/mL with anchor points (25.0 and 50.0 ng/mL). We confirmed the accuracy (% relative error, %RE) and precision (% coefficient of variation, %CV) of each assay using T-DM1 quality control (QC) samples prepared in rat serum (Table S2) and identified the sample dilution linearity on the measured concentration of the total trastuzumab and DM1-conjugated trastuzumab. As a result, the %RE between the concentration values from consecutive dilutions within a sample dilution series did not exceed 10.3% with the total trastuzumab and 7.3% with DM1-conjugated trastuzumab (Table S3). For both ELISAs, no hook effect (Table S4) and no potential interfering factors in rat serum were observed (Table S5). Moreover, we observed that T-DM1 in rat serum was stable at room temperature for up to 24 h, after storage for 1 month at −80 °C, and after five freeze/thaw cycles (Table S6). All the evaluated parameters satisfied the acceptance criteria of the European Medicines Agency’s guidelines on bioanalytical method validation [31].

### 3.2. Validation of LC–MS/MS for DM1

To determine the level of unconjugated DM1 in rat plasma, the LC–MS/MS assay was partially validated. The parameters evaluated during validation are described in Table 1. The results are provided in the supplemental information, Supplementary Material Tables S7–S9. The standard curve range was constructed from 2 to 400 ng/mL with a correlation coefficient of 0.9967. The accuracy (%RE) and precision (%CV) were within 6.5% and 10.6% of all plasma quality control levels (lower limit of quantitation (LLOQ), low quality control (LQC), middle quality control (MQC), and high quality control (HQC)), respectively (Table S8). No carry over peak was observed at the peak area of DM1. The DM1 in the rat plasma was stable at room temperature for up to 4 h, after storage for 1 month at −80 °C, and after three freeze/thaw cycles. Moreover, postpreparative stability was demonstrated by analysis of the extracted samples after being stored for 29 h in an autosampler at −4 °C (Table S9).

### 3.3. Characterization of Assay for ADC

We performed additional unique experiments for the evaluation of ADCs. As mentioned previously, the ELISA for the total trastuzumab was designed to detect conjugated and unconjugated T-DM1. To demonstrate the ability of the assay to quantify both the conjugated and unconjugated trastuzumab, T-DM1 (as a mixture of conjugated and unconjugated antibodies) and trastuzumab (as a completely unconjugated antibody) were added to the rat serum at different ratios and analyzed with T-DM1 calibration standards in the total trastuzumab ELISA (Table 2). The %RE for samples containing both T-DM1 and trastuzumab ranged from 3.6 to 19.0%. When only T-DM1 was added to the rat serum, the %RE was 4.5%; however, when only trastuzumab was analyzed with the T-DM1 calibration standards, the %RE was 31.5%. To support these results, we confirmed the characteristics of T-DM1 and trastuzumab with the total trastuzumab ELISA. The %RE range of the trastuzumab QC samples was from 2.0 to 21.5% with the trastuzumab standard curve. However, the %RE range of the trastuzumab QC samples was 23.3 to 56.1% with the T-DM1 standard curve, showing that the samples with higher concentrations of trastuzumab induced larger deviations from the nominal concentration of trastuzumab with the T-DM1 standard curve (Table 3). The results of these experiments showed that the %RE was higher with the unconjugated trastuzumab than with T-DM1 at all evaluated concentrations, suggesting that the PK change in T-DM1, including deconjugated metabolites, depended on which ELISA method was selected for the analysis of T-DM1.

### 3.4. PK Study of T-DM1 in Rats

**Figure 2:**
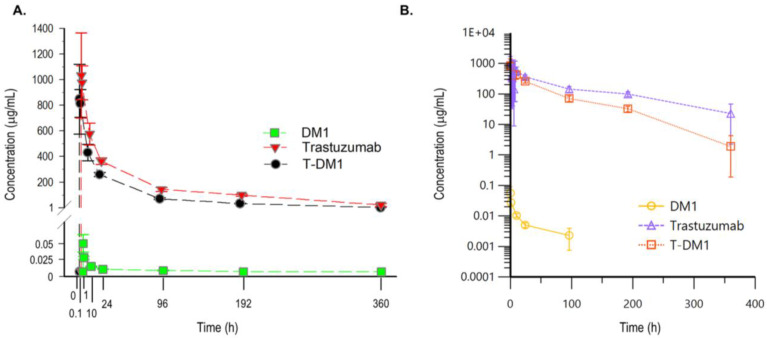
Pharmacokinetic profile of T-DM1 after a single intravenous injection of T-DM1 (20 mg/kg) in Sprague–Dawley rats. Sampling time points included predose (0 h) and 5 min, as well as 1, 10, 24, 96, 192, and 360 h post injection. Representative mean concentration–time graphs are depicted in (**A**) concentration–time curves and (**B**) Concentration in log scale–time curves. The concentration of DM1 in rat plasma was analyzed with LC–MS/MS (green closed square for (**A**), yellow open circle for (**B**)). T-DM1 was separately analyzed by targeting DM1 for the detection of DM1-conjugated trastuzumab (closed circle for (**A)**, red open square for (**B**)) or by targeting trastuzumab for the detection of total trastuzumab regardless of DM1 conjugation (red closed triangle for (**A)**, purple open triangle for (**B**)). The results are shown as the mean ± standard deviation.

The PK properties of T-DM1, total trastuzumab, and DM1 were evaluated. Figure 2 shows representative PK curves of T-DM1 administered to rats (20 mg/kg, single dose). Figure 2A shows the arithmetic concentration–time curves for total trastuzumab, conjugated trastuzumab, and DM1. The concentration in log scale–time curve for the three analytes is shown in Figure 2B. The relationship between the single-dose administration of T-DM1 and the PK parameters in the rat serum/plasma is presented in Table 4. The PK parameters were derived from noncompartmental analysis using WinNonlin. The results elucidated an approximately 1.2-fold enhancement in C_max_ upon the administration of T-DM1 into the rats, as C_max_ was 848.03 μg/mL for T-DM1 and increased to 1032.72 μg/mL for the total trastuzumab, as well as suggesting the appearance of deconjugated DM1. Moreover, the results revealed that T-DM1 exhibited an approximately two-fold increase in half-life for the total trastuzumab compared to T-DM1 or DM1, which showed the bioavailability of T-DM1 after administration. The steady-state volume of distribution of T-DM1 was similar to that of total trastuzumab, but the T-DM1 clearance was approximately 1.9-fold times faster than that of the total trastuzumab. The unconjugated DM1 had a significantly larger volume of distribution at the steady state (38,105 times larger) and a faster clearance than both T-DM1 and the total trastuzumab. Our data demonstrated that the low levels of DM1-conjugated trastuzumab in rats had similar patterns to those of monkey and human PK profiles [32,33].

### 3.5. Immunogenicity Assessment for T-DM1 Administered Rats

**Figure 3:**
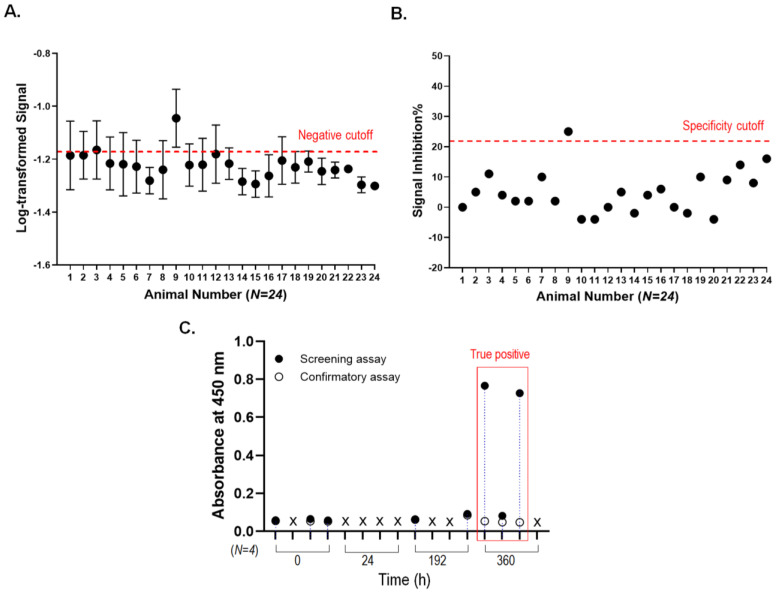
Immunogenicity assessment of T-DM1 in rats administered intravenously with a single dose. (**A**) Negative cutoff value and (**B**) specificity cutoff values were determined with the blank sera of 24 individual rats. The 95th percentile among the log-transformed values was defined as the negative cutoff, and the signal inhibition for specificity cutoff value was calculated as the mean %difference between T-DM1-untreated and T-DM1-treated individuals. (**C**) Screening and confirmatory assays were performed to discriminate ADA positivity against T-DM1. The previously set negative cutoff and specificity cutoff were applied. Following the screening assay, samples were categorized as either positive (marked as closed circles) or negative (marked as X). The positive samples were then subjected to confirmatory immunocompetition assay (marked as open circles) to differentiate between true and false positive results. True ADA-positive samples for T-DM1 are described in the red box.

To evaluate the immunogenicity of T-DM1 in a single-dose toxicity study in rats, we first developed an assay for a specific ADA, an anti-T-DM1 antibody, using a bridging ELISA. The developed ELISA method had high sensitivity (0.3 ng/mL), and the cutoff values used for the sample analysis were obtained from this ELISA method. The cutoff was determined as recommended by Mire-Sluis et al. [34], and 24 individual rat blank sera were measured with a bridging ELISA for the absorbance at the basal level to determine the negative cutoff value (Figure 3A). In two independent assays, samples in which the % coefficient of variation from the duplicate measurement values exceeded 25% and were outliers on a box plot analysis were excluded from the negative cutoff calculation. The negative cutoff value was defined as the 95th percentile among the log-transformed values because both the mean and log-transformed mean values were non-normally distributed according to the Shapiro–Wilk normality test (*p* > 0.05). The mean absorbance value of the naive blank sera was 0.067 (log value was −1.18), and the normalization factor was multiplied by 1.14 based on the assay validation experiments in rats. T-DM1 was treated with 24 individual rat blank sera to determine the signal inhibition at the basal level, and the specificity cutoff value was determined to be 22 (Figure 3B).

Serum samples obtained from T-DM1-administered rats were tested using the bridging ELISA as previously described. As a result of evaluating positivity in the screening assay, 8 out of the 16 collected samples showed absorbance values greater than the negative cutoff value. Following screening, a confirmation assay was conducted to determine whether potentially positive samples for T-DM1 antibodies that were detected by the initial screening assay were true or false positives. A total of three samples were identified as true positives, which showed signal inhibition values above the specificity cutoff, and all of them were at 360 h after injection. (Figure 3C).

## 4. Discussion

Current biotherapeutics are designed to be capable of multiple functions by increasingly complex structures. Since T-DM1 has the characteristics of small-molecule drugs and antibodies, there are unique challenges in developing bioanalytical methods for T-DM1 evaluation compared to developing methods for the evaluation of conventional therapeutic antibodies or small-molecule drugs. Since the small-molecule drug and the antibody of T-DM1 are critical to its activity, assays suited to quantitating these components are required [23]. Another report showed that ADCs with various DAR values performed differently in total and conjugated antibody assays [35]. Therefore, it is evidently important to evaluate two distinguished assays for T-DM1 or trastuzumab in vivo. Bioanalytical methods that have been validated in accordance with relevant regulatory and industry guidelines are critical for successful preclinical and clinical PK studies [31,36,37]. To support the PK study by providing accurate data, we validated two ELISAs for the antibody analysis of T-DM1: an ELISA designed to quantify total trastuzumab and an ELISA designed to quantify DM1-conjugated trastuzumab. All the typical parameters demonstrated for the ELISAs were acceptable. In addition, an LC–MS/MS assay was developed to measure the amount of free DM1. ADCs present unique challenges when developing bioanalytical methods for LBAs. The experiments unique to ADC demonstrated that there was a difference in recovery between T-DM1 and trastuzumab in the total trastuzumab ELISA. As Junttila et al. found, conjugating trastuzumab with DM1 does not affect the binding affinity of trastuzumab to HER2 [17]. On the other hand, ADCs with various DARs may have different binding affinities to the capture/detection reagents used in ELISAs [38]. As shown by our data in Table 2 and Table 3, total trastuzumab assays may theoretically have poor binding to T-DM1 with high DARs due to steric hindrance. Moreover, the conjugated trastuzumab ELISA may have poor binding to T-DM1 with low DARs, such as DAR 1, due to low avidity [24]. As a result, ADCs with different DAR values may not be uniformly quantified [38]. It was demonstrated that ADCs with various DARs performed differently in the total trastuzumab assay and conjugated trastuzumab assay [35,39,40]. Based on the results, the binding affinities of ADCs may be affected by conjugated DM1 in the total trastuzumab ELISA, which was developed in this study. To confirm the binding affinity of ADCs, further study is required to assess the impact of varying DARs on the accuracy of the total trastuzumab ELISA.

During preclinical development, the characterization of an ADC’s PK parameters is very important to predict its effects in humans. After administration, circulating levels of total trastuzumab and conjugated trastuzumab are expected to decrease over time. Our data established exposure–response relationships by evaluating PK parameters in rats. As expected, the total trastuzumab and conjugated trastuzumab concentrations at C_max_ were similar, but the C_max_ of trastuzumab was approximately 1.2-fold higher than that of T-DM1. These results were consistent with the appearance of free DM1 due to deconjugation from T-DM1. The C_max_ of T-DM1 in another rat study was lower than that of our study. The difference in these results might be due to the different sampling times between the studies. The first sampling point was 10 h, which was an insufficiently earlier time point to be considered an early phase sampling for PK [33]. In a previous monkey study, added to the lower dosing is the much faster clearance of T-DM1 [33]. The circulating half-life of total trastuzumab was longer than that of T-DM1. Taken together, the systemic exposure of T-DM1 is far less than that of trastuzumab. A study showed that ADCs with higher DARs cleared faster and were less tolerated than conjugates with lower DARs [41]. Although DM1 had the highest clearance, which was directly related to its molecular weight [42], the clearance of T-DM1 was slightly higher than that of the total trastuzumab. ADCs showed faster clearance when the mAb carrier was linked with a higher number of ligands. For example, T-DM1 was cleared more rapidly (3–4 fold) than unconjugated trastuzumab alone in both mice and humans when administered at similar doses [43,44]. The difference between the total trastuzumab and conjugated trastuzumab concentrations over time, in addition to the presence of DM1 in plasma, suggests that in vivo T-DM1 is deconjugated [13]. Although single-dose treatment was administered, the observed response rate in this study was comparable to the rates in other studies. For example, inotuzumab ozogamicin had similar profiles of the conjugated antibody and total antibody (conjugated antibody plus cleaved free antibody) to the profile of concentration versus time in the serum [45]. Similar PK results for the total trastuzumab and conjugated trastuzumab with low levels of DM1 suggest that T-DM1 is relatively stable in vivo. As a result of the concentration of the drug by a time-dependent concentration curve, the total trastuzumab had a terminal half-life approximately two times longer than that of conjugated trastuzumab and 1.9-fold lower clearance than conjugated trastuzumab. The results in this study are very similar to those in previous preclinical and clinical studies [13,32] and are consistent with findings in which the toxicity profile translated well from rat to monkey to human overall [33]. This evidence also showed that the three assays (total trastuzumab ELISA, DM1-conjugated trastuzumab ELISA, and LC–MS/MS assay for DM1) were successfully validated.

ADCs can cause an ADA response like other biological drugs. It is important to monitor and evaluate ADA responses to ADCs during both clinical and preclinical studies to understand the PK, safety, and efficacy over time [46]. We also tested the immunogenicity of the ADC using a bridge ELISA, which was optimized following the recommendations [34]. Since the bridging assay relies on the availability of both antigen-binding sites on ADAs, it can only detect drug-free ADAs when the free drugs are not complexed with ADAs produced after administration. In this study, we measured the ADA level at 360 h after some clearance occurred; thus, the bridging assay for ADA was susceptible to the samples [47]. After assessing the immunogenicity of the T-DM1-administered rats, only three samples at 360 h after injection were detected as true positives. There was no apparent effect of ADA on the PK parameters in the rats. In previous T-DM1 cynomolgus monkey studies [27], the ADA response did not affect the interpretation of PK from the studies. These results mean that the linker and payload of T-DM1 did not induce a high immune response in monkeys. Thus, it was confirmed that the immunogenicity rate of T-DM1 was low in both rats and monkeys. Although animal models are not always predictive of human immunogenicity [48], no significant effects of ADA on the PK or safety profiles of T-DM1 have been reported in clinical trials [32].

In conclusion, the results in this study suggested that the overall PK profile and immunogenicity translated well from rats to monkeys to humans. The bioanalytical methods optimized in this study provided a valuable approach to help understand the safety and efficacy of T-DM1. It will be important to continue using a variety of existing and new bioanalytical methods that provide appropriate information to help understand the safety and efficacy of various ADCs in preclinical and clinical studies. The data presented in this study establishes a systematic bioanalysis of ADCs with validated assays including drug stability in a matrix. The optimized ADA assays also improved the precise pharmacokinetic interpretation from quantification with a systematic bioanalysis process of ADCs. In addition, our bioanalytical experiences may support the development of bioanalytical methods on a case-by-case basis, using ADC characteristics to facilitate the future development of ADCs.

## Figures and Tables

**Table 1:** Validated bioanalytical parameters for quantification of T-DM1.

Parameters	DM1-Conjugated Trastuzumab	Total Trastuzumab	DM1
Analytical method	ELISA	ELISA	LC–MS/MS
Matrix	Serum	Serum	Plasma
Target molecule	DM1	trastuzumab	DM1
Minimum required dilution	1:1000	1:100	NA
Lower limit of quantitation (LLOQ)	100 ng/mL	100 ng/mL	2 ng/mL
Upper limit of quantitation (ULOQ)	6000 ng/mL	6000 ng/mL	400 ng/mL
Quantitative analysis model	4-parameter logistic regression model		1/x^2^-weighted linear regression model
Between-run accuracy (%RE)	−9.2–−4.4	−3.0–−0.8	−2.8–6.5
Between-run precision (%CV)	8.4–16.8	6.7–12.0	6.8–10.6

**Table 2:** Total trastuzumab ELISA results for samples containing both T-DM1 and trastuzumab.

	Ratio (T-DM1: Trastuzumab)				
	5:0	4:1	1:1	1:4	0:5
N. Con. (ng/mL)	1000.0	1000.0	1000.0	1000.0	1000.0
Mean ± SD	1045.5 ± 23.6	1036.5 ± 13.1	1159.6 ± 18.7	1189.9 ± 16.2	1315.2 ± 25.4
%CV	2.3	1.3	1.6	1.4	1.9
%RE	4.5	3.6	16.0	19.0	31.5

N. Con. (ng/mL): nominal concentration (ng/mL).

**Table 3:** Total trastuzumab ELISA results for trastuzumab samples evaluated with T-DM1 curve or trastuzumab curve.

	Trastuzumab with Trastuzumab Standard Curve					Trastuzumab with T-DM1 Standard Curve				
	LLOQ	LQC	MQC	HQC	ULOQ	LLOQ	LQC	MQC	HQC	ULOQ
N. Con. (ng/mL)	100.0	300.0	1000.0	3000.0	6000.0	100.0	300.0	1000.0	3000.0	6000.0
Mean ± SD	102.0 ± 2.2	306.9 ± 10.9	1092.7 ± 31.4	3244.8 ± 85.3	7289.7 ± 271.7	126.4 ± 2.7	369.8 ± 12.8	1274.3 ± 36.1	3830.4 ± 105.8	9367.0 ± 410.8
%CV	2.1	3.6	2.9	2.6	3.7	2.1	3.4	2.8	2.8	4.4
%RE	2.0	2.3	9.3	8.2	21.5	26.4	23.3	27.4	27.7	56.1

N. Con. (ng/mL): nominal concentration (ng/mL).

**Table 4:** PK parameters following single-dose administration of T-DM1 (20 mg/kg) in rats.

Parameters	DM1	Total Trastuzumab	T-DM1
T_max_ (min)	5	5	5
T_1/2 terminal_ (h)	1.89	3.69	1.96
C_max_ (μg/mL) *	56.40 ± 17.53 ^a^	1032.72 ± 331.63	848.03 ± 271.89
AUC_0-360_ (day·μg/mL) *	24.49 ± 1.74 ^b^	2280.29 ± 87.67	1291.40 ± 52.07
AUC_0-inf_ (day·μg/mL)	30.82 ^b^	2401.55	1296.76
Cl (mL/day/kg)	1126.65	8.40	15.36
Vss (mL/kg)	1430091.52	37.53	37.85

* The results are shown as the mean ± standard error. ^a^ Units are ng/mL; ^b^ units are day·ng/mL; T_max_, time of maximum concentration; T_1/2 terminal_, terminal half-life; C_max_, maximum observed concentration; AUC_0-360_, area under the concentration versus time curve from 0 to 360 h; AUC_0-inf_, area under the concentration versus time curve from time 0 to infinity; Cl, clearance; Vss, volume of distribution at steady state.

## Data Availability

Not applicable.

## Supplementary Materials

The following supporting information can be downloaded at https://www.mdpi.com/article/10.3390/pharmaceutics15030756/s1: Table S1: Calibration Standards of ELISAs for T-DM1 in Rat; Table S2: Accuracy and Precision of ELISAs for T-DM1 in Rat; Table S3: Dilution Linearity of ELISAs for T-DM1 in Rat; Table S4: Hook Effect of ELISAs for T-DM1 in Rat; Table S5: Matrix Effects (Selectivity) of ELISAs for T-DM1 in Rat; Table S6: Stability of ELISAs for T-DM1 in Rat; Table S7: Calibration Standards of LC–MS/MS for DM1 in Rat; Table S8: Accuracy and Precision of LC–MS/MS for DM1 in Rat; Table S9: Stability of LC–MS/MS for DM1 in Rat.
