# Supplementary material for: Implementation of Systematic Bioanalysis of Antibody–Drug Conjugates for Preclinical Pharmacokinetic Study of Ado-Trastuzumab Emtansine (T-DM1) in Rats
Source: Pharmaceutics. 2023 Feb 24;15(3):756. doi: 10.3390/pharmaceutics15030756 (PMC10056844; doi:10.3390/pharmaceutics15030756)
Supplement: Supplementary file 1 [file pharmaceutics-15-00756-s001.zip › pharmaceutics-2159884-supplementary.pdf]

Table S1. Calibration Standards of ELISAs for T-DM1 in Rat

	T-DM1 (Total Trastuzumab ELISA) (N=10)			T-DM1 (DM1-Conjugated Trastuzumab ELISA) (N=8)			Trastuzumab (Total Trastuzumab ELISA) (N=1)	
Nominal Concentration (ng/mL)	Mean ± SD	%CV	%RE	Mean ± SD	%CV	%RE	Mean	%RE
25.0[a]	23.2 ± 6.2	26.8	-7.0	24.5 ± 8.6	35.0	-1.9	27.1	8.4
50.0[a]	49.0 ± 1.5	3.1	-2.0	51.3 ± 5.9	11.5	2.6	49.4	-1.3
100	101.2 ± 6.2	6.1	1.2	98.2 ± 7.7	7.9	-1.8	96.3	-3.7
250	253.6 ± 8.5	3.4	1.4	252.5 ± 11.5	4.6	1.0	250.6	0.3
500	497.0 ± 7.0	1.4	-0.6	496.4 ± 13.1	2.6	-0.7	498.8	-0.2
1000	1005.3 ± 28.4	2.8	0.5	995.5 ± 11.8	1.2	-0.4	1023.9	2.4
2000	1967.2 ± 37.6	1.9	-1.6	2034.1 ± 74.1	3.6	1.7	1935.3	-3.2
4000	4231.3 ± 255.1	6.0	5.8	3928.8 ± 198.0	5.0	-1.8	4114.8	2.9
6000	5783.4 ± 313.8	5.4	-3.6	6061.3 ± 170.1	2.8	1.0	5927.5	-1.2

[a]: Anchor point; acceptance criteria does not apply.

Table S2. Accuracy and Precision of ELISAs for T-DM1 in Rat

		Total Trastuzumab ELISA (N=20, 8 runs)				DM1-Conjugated Trastuzumab ELISA (N=18, 7 runs)			
Quality Control	Nominal Concentration (ng/mL)	Mean ± SD	%CV	%RE	Total Error (|%RE| +%CV)	Mean ± SD	%CV	%RE	Total Error (|%RE| +%CV)
LLOQ	100	97.6 ± 9.0	9.2	-2.4	11.6	95.5 ± 11.5	12.0	-4.5	16.5
LQC	300	294.9 ± 19.8	6.7	-1.7	8.4	286.7 ± 48.1	16.8	-4.4	21.2
MQC	1000	991.8 ± 81.9	8.3	-0.8	9.1	907.6 ± 81.4	9.0	-9.2	18.2
HQC	3000	2921.3 ± 333.2	11.4	-2.6	14.0	2733.9 ± 285.8	10.5	-8.9	19.3
ULOQ	6000	5818.1 ± 699.2	12.0	-3.0	15.0	5585.0 ± 470.8	8.4	-6.9	15.3

Table S3. Dilution Linearity of ELISAs for T-DM1 in Rat

		Total Trastuzumab ELISA			DM1-Conjugated Trastuzumab ELISA		
Dilution factor	Nominal Concentration (ng/mL)	Mean ± SD	%CV	%RE	Mean ± SD	%CV	%RE
1:200	1000000	896741 ± 142571	15.9	-10.3	952347 ± 64966	6.8	-4.8
1:1000	1000000	1025909 ± 66696	6.5	2.6	937656 ± 28807	3.1	-6.2
1:5000	1000000	954359 ± 79046	8.3	-4.6	926524 ± 26398	2.8	-7.3

Table S4. Hook Effect of ELISAs for T-DM1 in Rat

		Total Trastuzumab ELISA	DM1-Conjugated Trastuzumab ELISA
Dilution Factor	Nominal Concentration After Dilution (ng/mL)	Observed Concentration (ng/mL)	Observed Concentration (ng/mL)
No Dilution	1000000	>ULOQ	>ULOQ
1:10	100000	>ULOQ	>ULOQ
1:100	10000	>ULOQ	>ULOQ

>ULOQ: Raw OD result is greater than raw OD result of the ULOQ in calibration standard (>6000.0 ng/mL)

Table S5. Matrix Effects (Selectivity) of ELISAs for T-DM1 in Rat

	Total Trastuzumab ELISA			DM1-Conjugated Trastuzumab ELISA		
	Blank	LLOQ		Blank	LLOQ	
Nominal Concentration (ng/mL)	0.0	100.0		0.0	100.0	
Individual unit ID	Observed Concentration (ng/mL)	Observed Concentration (ng/mL)	%RE	Observed Concentration (ng/mL)	Observed Concentration (ng/mL)	%RE
1	BLQ	100.1	0.1	BLQ	93.9	-6.1
2	BLQ	110.2	10.2	BLQ	95.9	-4.1
3	BLQ	78.0	-22.0	BLQ	71.8	-28.2
4	BLQ	108.3	8.3	BLQ	108.8	8.8
5	BLQ	97.1	-2.9	BLQ	97.7	-2.3
6	BLQ	107.4	7.4	BLQ	95.8	-4.2
7	BLQ	109.1	9.1	BLQ	90.1	-9.9
8	BLQ	119.0	19.0	BLQ	96.1	-3.9
9	BLQ	89.0	-11.0	BLQ	98.6	-1.4
10	BLQ	105.1	5.1	BLQ	68.2	-31.8

BLQ: Below the Limit of Quantification (<100.0 ng/mL)

Table S6. Stability of ELISAs for T-DM1 in Rat

Table S6-1. Total Trastuzumab

	Room temperature stability (24 hours)		Freeze/Thaw stability (5 cycles)		Long-term stability (-80°C, 31 days)	
Nominal Concentration (ng/mL)	300.0	3000.0	300.0	3000.0	300.0	3000.0
Mean ± SD	303.4 ± 10.0	3030.0 ± 121.7	344.9 ± 9.4	3107.1 ± 79.4	338.7 ± 13.2	3282.7 ± 69.7
%CV	3.3	4.0	2.7	2.6	3.9	2.1
%RE	1.1	1.0	15.0	3.6	12.9	9.4

Table S6-2. DM1-Conjugated Trastuzumab

	Room temperature stability (24 hours)		Freeze/Thaw stability (5 cycles)		Long-term stability (-80°C, 32 days)	
Nominal Concentration (ng/mL)	300.0	3000.0	300.0	3000.0	300.0	3000.0
Mean ± SD	242.5 ± 11.2	2933.8 ± 195.9	262.8 ± 9.0	2834.6 ± 347.0	285.3 ± 6.4	2541.2 ± 57.2
%CV	4.6	6.7	3.4	12.2	2.2	2.3
%RE	-19.2	-2.2	-12.4	-5.5	-4.9	-15.3

Table S7. Calibration Standards of LC–MS/MS for DM1 in Rat

	Calibration Standards [a]							
Nominal Concentration (ng/mL)	2.0	5.0	10.0	20.0	50.0	150.0	250.0	400.0
Mean ± SD	2.0 ± 0.1	5.1 ± 0.4	9.7 ± 0.8	19.5 ± 1.4	50.3 ± 4.2	157.8 ± 11.4	253.3 ± 12.7	390.7 ± 19.6
%CV	4.1	7.4	8.6	7.3	8.3	7.2	5.0	5.0
%RE	0.0	1.7	-3.3	-2.4	0.6	5.2	1.3	-2.3

[a] N=7/concentration

Table S8. Accuracy and Precision of LC–MS/MS for DM1 in Rat

Quality Control [a]	LLOQ	LQC	MQC	HQC
Nominal Concentration (ng/mL)	2.0	5.0	100.0	300.0
Mean ± SD	1.9 ± 0.2	5.1 ± 0.4	105.6 ± 8.4	319.4 ± 21.7
%CV	10.6	8.6	7.9	6.8
%RE	-2.8	2.4	5.6	6.5

[a] Total 3 runs were performed for accuracy and precision (N=6/concentration/run).

.

Table S9. Stability of LC–MS/MS for DM1 in Rat

	Room temperature stability (4 hours)		Freeze/Thaw stability (3 cycles)		Long-term stability (-80℃, 7 days)		Post-preparative stability (4℃, 29 hours)	
Nominal Concentration (ng/mL)	5.0	300.0	5.0	300.0	5.0	300.0	5.0	300.0
Mean ± SD	4.3 ± 0.1	310.1 ± 5.2	4.5 ± 0.2	305.3 ± 14.8	4.6 ± 0.3	301.1 ± 22.8	4.4 ± 0.2	331.4 ± 22.6
%CV	2.7	1.7	4.6	4.8	6.2	7.6	3.9	6.8
%RE	-13.3	3.4	-9.3	1.8	-7.3	0.4	-12.0	10.5
